# Implementing occupational therapy quality indicators in a large healthcare system: lessons from a 5-year performance monitoring study

**DOI:** 10.1093/intqhc/mzag081

**Published:** 2026-06-05

**Authors:** Khawla Loubani, Orit Segev-Jacubovski, Doron Comaneshter, Arnon D Cohen, Ariela Zur, Doron Netzer

**Affiliations:** Department of Occupational Therapy, Ben-Gurion University of the Negev, Beer Sheva, Israel; Clalit Health Services, Tel Aviv, Israel; Clalit Health Services, Tel Aviv, Israel; Department of Occupational Therapy, Ariel University, Ariel, Israel; Clalit Health Services, Tel Aviv, Israel; Department of Quality Measurements and Research, Clalit Health Services, Tel Aviv, Israel; Faculty of Health Sciences, Ben-Gurion University of the Negev, Beer Sheva, Israel; Clalit Health Services, Tel Aviv, Israel; Adelson School of Medicine, Ariel University, Ariel, Israel; Community Medical Services Division, Clalit Health Services, Tel Aviv, Israel

**Keywords:** rehabilitation service, service delivery, performance measurement

## Abstract

**Background:**

Performance measurement tools, such as quality indicators, shape how services are organized, delivered, and evaluated; they are central to monitoring and improving health system performance. This study examines 5 years of implementing occupational therapy quality indicators within a large public healthcare organization and assesses how structured performance measurement can support routine monitoring, accountability, and quality improvement in occupational therapy service delivery.

**Method:**

We conducted this retrospective, repeated cross-sectional study within Clalit Health Services, Israel’s largest public healthcare organization, serving more than 4.9 million members across nine districts. We analysed performance trends (2021–5) for five occupational therapy quality indicators implemented system-wide: children–parental conversations, children-treatment interruption, frail older adults, stroke, and hip fracture. Data were extracted from electronic health records and administrative databases. The analytic sample included all Clalit Health Services members eligible for at least one occupational therapy quality indicator during the study period. Performance for each occupational therapy quality indicator was calculated as numerator/denominator * 100 national-level longitudinal trends and subgroup associations were analysed.

**Results:**

Four of the five occupational therapy-quality indicators improved significantly over time. From 8.1% to 3.7%, occupational therapy service delivery to frail older adults increased from 51.8% to 77.4%, and occupational therapy after stroke increased from 52.2% to 59.9% (*P *< .001 for all). The hip fracture indicator showed no statistically significant overall change, increasing slightly from 71.3% to 71.8% (*P *= .706), despite fluctuations during the monitoring period. Methodological changes to the denominator definition highlighted the sensitivity of performance metrics to operational definitions. Subgroup analyses further identified socio-demographic and geographic variation in occupational therapy quality indicator performance, particularly by age, socio-economic position, ethnicity, and peripherality, although patterns differed across indicators.

**Conclusions:**

Implementing occupational therapy quality indicators within a nationwide community rehabilitation system enabled routine longitudinal monitoring of access, timeliness, family engagement, continuity of care, and service consistency. These indicators provided a structured framework for identifying service gaps, improving national-level visibility, and informing organizational decision-making. The findings support the feasibility and value of profession-specific quality indicators as a scalable model for performance monitoring and quality improvement in rehabilitation and allied health services. Future research should link occupational therapy quality indicator performance to patient-level functional, participation, and quality-of-life outcomes and examine geographic and socio-demographic inequities in service delivery.

## Graphical abstract

**Graphical Abstract:**
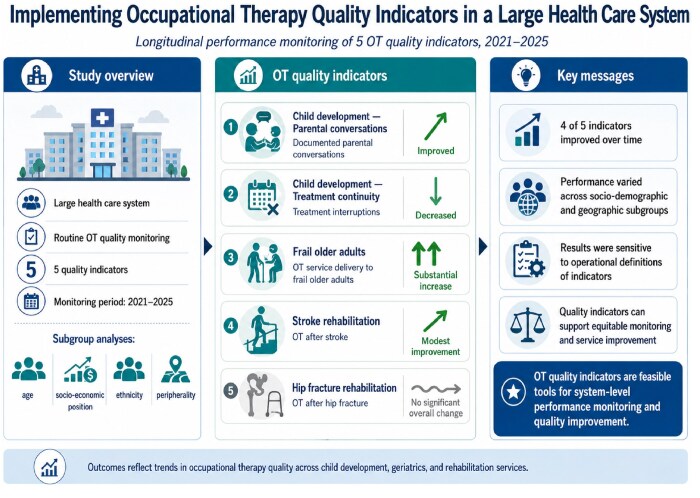

## Introduction

Performance measurement tools, such as quality indicators (QIs), are central to monitoring and improving health system performance, shaping how services are organized, delivered, and evaluated [1, 2]. QIs are explicit, measurable items referring to care structures, processes, or outcomes, usually expressed as numerator/denominator (score indicates quality) [3, 4]. In universal healthcare systems, they support monitoring of access, equity, service consistency, and adherence to defined care standards [5]. However, profession-specific QIs are needed to assess whether occupational therapy (OT) services are delivered consistently and equitably across populations and geographic regions.

In Israel, OT is provided primarily through community-based services within the publicly funded healthcare system [6]. Clalit Health Services (CHS), the country’s largest healthcare organization, serves over 4.9 million members (approximately 53% of the population), representing diverse age, gender, socio-economic, ethnic, and geographic groups. It delivers OT services across multiple clinical areas, including paediatrics, geriatrics, orthopaedics, and neurological rehabilitation. To support systematic performance monitoring, the OT sector within CHS has developed and monitored five OT-QIs since 2021, addressing access, timeliness, family engagement, and continuity of care.

Although QIs are well established in medical practice and healthcare management, their systematic use within OT remains limited [4, 6, 7]. This gap is important because OT addresses complex, functional, participation-oriented, and context-dependent outcomes that conventional biomedical indicators may not capture. OT-specific QIs can therefore strengthen accountability, service monitoring, and quality improvement. As evidence on OT-QIs in community healthcare remains scarce, with no previous studies in Israel, this study describes the development and monitoring of OT-QIs within CHS and analyses longitudinal performance across population subgroups. We examined 5 years of system-wide performance data, aiming to (i) describe the CHS OT-QI development process; (ii) examine longitudinal trends in OT-QI performance; and (iii) examine associations between OT-QI performance and demographic, socio-economic, ethnic, and geographic characteristics.

## Materials and methods

### Design and setting

This retrospective, descriptive, repeated, cross-sectional study analysed 5 years (2021–5) of performance data for five OT-QIs implemented across paediatric, geriatric, orthopaedic, and neurological rehabilitation settings within CHS.

### Development and selection of OT-QIs

The five OT-QIs were developed through a structured process involving CHS clinical experts, service managers, and quality improvement professionals. Steps recommended by the modified RAND method [8] and Leland *et al.* [9] guided the OT-QI selection and development. We selected this method because it combines evidence synthesis with expert consensus, ensuring the developed OT-QIs are scientifically valid and practically applicable in the CHS context. This article focuses on the first four phases of this process: measuring development, specification, and evaluation and monitoring implementation through performance-trend analysis.

#### Phase 1: OT-QI development

CHS convened a national expert panel of 30 OT professionals representing child development, geriatrics, physical rehabilitation, mental health, and home care. Within CHS, OT services are delivered by licensed occupational therapists with at least a bachelor’s degree and Ministry of Health licensure. The Ministry of Health guidelines, relevant literature, and CHS strategic priorities informed the topic selection. This process, conducted between 2012 and 2015, prioritized OT-QIs based on stakeholder needs and existing evidence-based clinical knowledge. Initially, it focused on child development OT-QIs and later expanded to adults, including stroke, hip fracture, and frailty. The expert panel defined each OT-QI’s target population, setting, intervention type, objectives, operational definitions, and calculation methods.

#### Phase 2: OT-QI specification

The expert panel defined essential parameters for each OT-QI, including target population characteristics, service settings, intervention types, objectives, operational definitions, and calculation methods. Consensus was achieved through iterative discussions following the modified RAND [8] method. These efforts aimed to align the OT-QIs with CHS’s broader strategic priorities, including waiting-time reduction and equitable service availability.

#### Phase 3: Determining the evaluation method

This phase assessed whether each OT-QI could be reliably identified and extracted from CHS’s electronic medical record (EMR) system. Each indicator was linked to predefined CHS EMR treatment codes documenting relevant OT interventions. This feasibility step confirmed the operationalization and extractability of all OT-QIs from routine EMRs.

#### Phase 4: Implementation

Implementation focused on establishing standardized documentation practices to enable performance monitoring. Occupational therapists documented predefined service codes for delivered interventions in the EMRs. Training sessions supported implementation by promoting accurate, consistent documentation. Documentation of intervention codes became part of routine clinical practice, enabling systematic monitoring of OT-QI performance. After CHS senior leadership provided final approval of the OT-QIs, the indicators were incorporated into the organization’s strategic quality framework.

### OT-QI description

The final five OT-QIs covered four clinical populations and key service delivery domains: access, timeliness, continuity of care, and family engagement. Table 1 presents the definitions, target populations, numerators, denominators, and scoring methods. Performance scores were calculated as numerator/denominator × 100; the direction of better performance depended on the indicator.

**Table 1:** Clalit Health Services OT-QIs: population served, aims, denominator, numerator, and performance score meaning.

QI	Description and rationale	Service type, setting	Aim	Denominator (target population)	Numerator	Performance score meaning
Parental conversations	Percentage of children receiving OT services, whose OT provider documented conversations with parents regarding treatment goals and progress; assesses whether formal parental conversations took place during eight OT sessions in community or clinical settings, at the end of a session, in-person, online, or by phone. Aims to improve OT–parent communication. Children referred to OT for developmental, sensory-motor, graphomotor, neurodevelopmental, or participation-related functional difficulties. Studies have shown that consistent OT–parent communication is essential for effective paediatric care; it supports treatment continuity [10–12], promotes parental engagement, improves adherence, and reduces treatment interruptions [13]. Children whose parents were more engaged and consistently attended therapy sessions made significantly greater developmental gains than those with irregular attendance [14]. Accordingly, recent paediatric OT evidence emphasizes family-centred, parent-mediated, and coaching-based interventions as core practice approaches [15].	Conversations between the OT practitioner and children’s parents, in-clinic or remote	Increase OT–parent communication, increase parental involvement in the intervention process, align expectations, reinforce therapeutic goals, and provide guidance for home practice	Children aged 0–9 years with at least eight OT visits, starting from their first visit during the measurement period	Clients (of denominator) with documented parent–OT practitioner conversations during the eight OT visits in the measurement period	Higher = better
Treatment interruption	Percentage of children with unplanned treatment interruptions exceeding 90 days; assesses whether treatment was interrupted (at least two consecutive missed appointments) over eight OT sessions. Treatment continuity is crucial in paediatric OT because missing two or more sessions can hinder skill acquisition and delay progress. Evidence from short-term OT interventions with preschoolers with developmental disabilities demonstrated significant developmental gains but only with consistent attendance. Missed sessions weakened these effects, highlighting that consistent participation is essential for achieving expected outcomes [13, 14].	At least two consecutive missed appointments	Decrease missed OT appointments during treatment and promote continuity of care	Children aged 0–9 years with at least eight OT visits, starting from their first visit during the measurement period	Clients (of denominator) with documented parent–OT practitioner conversations during the eight OT visits in the measurement period	Lower = better
Frail older adults	Percentage of frail older adults (aged 65+) who received at least one OT assessment or intervention during the measurement period; assesses whether OT services were provided to older adults at risk of functional decline following a physician’s medical diagnosis in the community or in a hospital [16, 17]. Increased OT involvement with this population could significantly enhance functional outcomes: OT interventions are particularly effective when delivered within a home or community-based framework, enabling individualized approaches to improving daily living activities, social participation, mobility, and fall prevention.	Individuals diagnosed as frail older adults; evaluation or intervention, in-clinic, home, or remote	Increase OT involvement among older adults at risk for functional decline within the community setting	Clients 65+ diagnosed as frail older adults by a physician who received OT evaluation/intervention during the measurement period (1 year) or 3 months after diagnosis	Clients (of denominator) who received an OT assessment or intervention during the measurement period or within 3 months after diagnosis	Higher = better
Individuals after stroke^a^	Percentage of stroke survivors who received OT services within 30 days of hospital discharge; aims to improve early OT involvement in community settings. Early interventions with this population address sensory-motor, perceptual-cognitive, and daily living skills and improve patients’ functional recovery and daily participation [18–20]. Following hospital discharge, OT interventions enhance reintegration into work and social activities, while offering counselling and support to families and caregivers. In randomized trials like the 2024 EOTIPS [18], early OT interventions were shown to significantly improve quality of life, functional independence, and perceptual-cognitive skills, reduce depression [18], and bolster social and work participation post-stroke [20]. Community-based rehabilitation soon after discharge supports rapid recovery and sustained engagement in life roles [20].	Individuals after stroke; evaluation or intervention, in-clinic, home, or remote	Improve early OT involvement among individuals after stroke within hospital or community settings	Clients with a primary diagnosis of stroke, discharged from hospital to home, who received OT within 1 year after discharge	Clients (of denominator) who received OT within 1 month after hospital discharge	Higher = better
Individuals after hip fracture^a^	Percentage of patients who received OT services within 30 days of hospital discharge for a hip fracture caused by a fall, trauma, or pathological fracture. Hip fractures are a significant public health issue, especially among older adults. They are primarily caused by falls and exacerbated by sensory, physical, or cognitive impairments [21]. Fractures adversely affect many aspects of daily living, leading to decreased participation, functional dependence, reduced quality of life, and even mortality [21, 22]. Early OT interventions are vital for preserving functional independence and quality of life, addressing mobility, home environment safety, pain management, cognitive and psychological support, fall prevention, use of assistive devices, patient education, and activity enhancement [22].	Individuals after hip fracture; evaluation or intervention, in-clinic, home, or remote	Improve early OT involvement among individuals after hip fracture	Clients with a primary diagnosis of hip fracture who underwent hip fracture surgery and who received OT within 1 year after discharge	Clients (of denominator) who received OT within 1 month after hospital discharge	Higher = better

Numerator = number of clients of the denominator meeting specific criteria within the assessed QI.

a For January to June 2021, the inclusion period was 1 year; from July 2021 onward, it was expanded to 2 years. For all other OT-QIs, the measurement period covered the past year.

### Data sources and extraction

We retrieved OT-QI data from the CHS EMRs. This comprehensive computerized database receives continuous real-time input from medical, pharmaceutical, and administrative systems, facilitating large-scale epidemiological analyses. The data undergo continuous diagnostic validation through automated checks (e.g. cross-verification of diagnoses from multiple sources) and direct verification by the treating physicians. The study population included all CHS clients (any age) who met the inclusion criteria for each OT-QI between January 2021 and December 2025. The database automatically excluded clients who had died, transferred to another healthcare organization, were new members (<1 year), or were active-duty soldiers (whose medical care is provided by the military).

Data are presented from January 2021 onwards because COVID-19 disrupted earlier implementation. By 2021, service processes had stabilized nationwide. The anonymized dataset covered all nine CHS districts and contained no personally identifiable information.

### Indicator calculation/performance measurement

The performance score for each OT-QI is calculated as numerator/denominator *100, where the *numerator* is the number of clients within the denominator who meet the performance criterion defined for each OT-QI according to EMRs, and the *denominator* represents the target population (all clients who meet the OT-QI inclusion criteria within the defined measurement window). Scores can be computed at multiple aggregation levels: by OT department, across departments within a district, and nationwide. In this article, we report nationwide performance scores and examine associations between OT-QI performance and demographic, socio-economic, ethnic, and geographic characteristics. Table 1 presents the detailed parameters (denominator, numerator, and scoring).

### Data analysis

We used descriptive statistics to present the sample characteristics. Categorical variables were presented as frequencies and percentages, including sample size, gender distribution (men, women), socio-economic status (SES; low, low-moderate, moderate-high, or high), age group, population sector, and peripherality index.

The relative change (delta) from the first measurement (January 2021) to the last measurement (December 2025) was calculated for each OT-QI as Δ = (Dec 2025 value − Jan 2021 value)/Jan 2021 value.

For child development QIs, age groups reflected allocated care hours: 0–2 years (unlimited sessions), 3–5 years (up to 27 sessions), and 6–9 years (up to nine sessions). For adult OT-QIs, participants were grouped as 18–44, 45–64, 65–74, and ≥75 years, according to Park *et al.* [16].

The SES, population sector, and peripherality were derived from patients’ residential addresses reported in EMRs. The SES was based on scores assigned by the Israeli Central Bureau of Statistics [23] and updated by POINTS Location Intelligence Company [24] using current socio-demographic and commercial data. The SES scores ranged from 1 to 10 and were categorized as low (1–3), low-moderate (4–5), moderate-high (6–7), and high (8–10). The population sector was categorized as secular Jewish, Orthodox Jewish, and Arab, based on residential area classification. The peripherality index of local authorities was categorized as peripheral (1–5), intermediate (6), and central (7–10).

Chi-square tests were used to examine associations between each OT-QI performance score and gender, age group, SES, population sector, and peripherality index.

Separate multivariable logistic regression models were fitted for each OT-QI to identify demographic and social characteristics associated with QI achievement, rather than to infer causal effects of the QI implementation program. In each model, the dependent variable was the OT-QI performance score. Gender, age group, SES, population sector, and peripherality index were entered simultaneously as explanatory variables. Results were reported as adjusted odds ratios with 95% confidence intervals. Statistical analysis was performed using IBM SPSS (version 29.0) [25]. A two-sided *P-*value of <.05 was considered statistically significant.

## Results

### Overall OT-QI monitoring trends

The OT-QIs captured a broad range of child development and adult populations across diverse geographic and socio-demographic contexts. Across all OT-QIs, the total target population (denominator) increased from 11 508 individuals (2852 children) in January 2021 to 19 898 individuals (3991 children) in December 2025. Over the same period, the number of individuals meeting the predefined performance criteria (numerator) increased from 5080 (1130 children) to 11 260 (2605 children).

**Figure 1:**
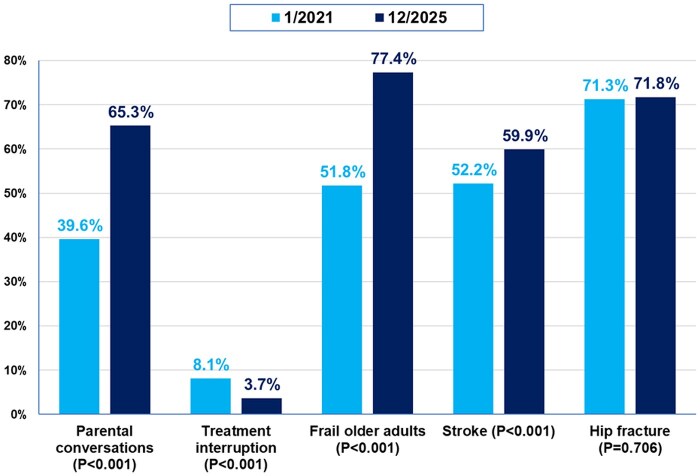
Changes in occupational therapy quality indicator performance scores (%) between January 2021 and December 2025. *Note*. Significant changes over time were observed for four indicators (*P* < .001): parental conversations increased, children’s treatment interruptions decreased, occupational therapy guidance for frail older adults increased, and occupational therapy after stroke increased. The hip fracture indicator showed no statistically significant change (*P* = .706).

Figure 1 presents changes in OT-QI rates between January 2021 and December 2025. Four of the five indicators improved significantly over time, including an increase in parental conversations, OT guidance for frail older adults, and OT after stroke, and a decrease in treatment interruptions. The hip fracture QI remained stable, with no statistically significant change.

### Longitudinal OT-QI performance trends

Table 2 presents the overall changes in OT-QI performance scores across the study period. Detailed longitudinal data are provided in Supplementary Tables S1 and S2. From January 2021 to December 2025, parental conversations increased from 39.6% to 65.3%, and treatment interruptions decreased from 8.1% to 3.7%, representing relative changes of 65% and −55%, respectively (*P *< .001 for both). Performance also increased significantly for frail older adults, from 51.8% to 77.4%, and for stroke, from 52.2% to 59.9% (*P *< .001 for both). In contrast, the hip fracture OT-QI remained stable, with no statistically significant change (*P *= .706).

**Table 2:** Changes in OT-QI performance scores between January 2021 and December 2025.

OT-QI	January 2021 *n*/*N* (%) [95% CI]	December 2025 *n*/*N* (%) [95% CI]	*P*-value	Relative change
Parental conversations	1130/2852 (39.6%) [37.31–41.93]	2605/3991 (65.3%) [62.77–67.78]	<.001	64.7%
Treatment interruption	232/2852 (8.1%) [7.09–9.18]	147/3991 (3.7%) [3.09–4.28]	<.001	−54.7%
Frail older adults	2070/3999 (51.8%) [49.53–53.99]	4305/5564 (77.4%) [75.06–79.68]	<.001	49.5%
Stroke	1484/2842 (52.3%) [49.56–54.87]	3106/5184 (59.9%) [57.81–62.02]	<.001	14.7%
Hip fracture	1294/1815 (71.3%) [67.41–75.18]	3702/5159 (71.8%) [69.45–74.07]	0.70	0.6%

*Note*. CI = confidence interval. Relative change (delta) was calculated between January 2021 and December 2025.

### Associations between OT-QI performance and demographic and social characteristics

Tables 3 and 4 present the associations between OT-QI performance and demographic and social characteristics across the five indicators, based on the univariate and multivariable analyses. Table 3 summarizes the child development indicators, and Table 4 presents the adult and older adult indicators.

**Table 3:** Associations between child development OT-QI performance and demographic and social characteristics (December 2025).

Variable	Category	Parental conversations *n*/*N* (%)	Univariate *P*-value	Parental conversations adjusted OR (95% CI)	Multivariable *P*-value	Treatment interruption *n*/*N* (%)	Univariate *P*-value	Treatment interruption adjusted OR (95% CI)	Multivariable *P*-value
Gender	Male	1886/2863 (65.9)	.200	Reference	.194	94/2863 (3.3)	.030	Reference	.110
	Female	719/1128 (63.7)		0.91 (0.78–1.05)		53/1128 (4.7)		1.34 (0.93–1.92)	
Age group (years)	0–2	269/413 (65.1)	<.001	Reference	<.001	9/413 (2.2)	.230	Reference	.200
	3–5	1824/2680 (68.1)		1.15 (0.92–1.44)	.210	103/2680 (3.8)		1.87 (0.94–3.75)	.080
	6–9	512/898 (57.0)		0.72 (0.56–0.92)	.010	35/898 (3.9)		1.69 (0.79–3.64)	.180
SES	Low	306/447 (68.5)	.002	Reference	<.001	30/447 (6.7)	.001	Reference	.01
	Low–medium	771/1237 (62.3)		0.88 (0.67–1.14)	.330	41/1237 (3.3)		0.46 (0.25–0.83)	.01
	Medium–high	866/1343 (64.5)		0.96 (0.73–1.28)	.800	48/1343 (3.6)		0.49 (0.25–0.93)	.03
	High	562/802 (70.1)		1.28 (0.94–1.74)	.120	19/802 (2.4)		0.29 (0.13–0.61)	.001
Ethnicity	Secular Jewish	2157/3334 (64.7)	.20	Reference	.100	114/3334 (3.4)	.010	Reference	.56
	Arab	184/274 (67.2)		1.19 (0.87–1.64)	.270	19/274 (6.9)		1.17 (0.59–2.31)	.660
	Orthodo Jewish	264/383 (68.9)		1.31 (1.01–1.69)	.040	14/383 (3.7)		0.78 (0.41–1.50)	.460
Peripherality index	Peripheral	757/1267 (59.7)	<.001	Reference	<.001	50/1267 (3.9)	.010	Reference	.008
	Medium	449/619 (72.5)		1.62 (1.30–2.01)	<.001	10/619 (1.6)		0.47 (0.23–0.96)	.040
	Centre	1383/2083 (66.4)		1.21 (1.04–1.42)	.010	87/2083 (4.2)		1.32 (0.90–1.94)	.160

Reference categories: male, age 0–2 years, low socio-economic position, secular Jewish, and peripheral regions. CI, confidence interval; OR, odds ratio; SES, socio-economic status.

**Table 4:** Associations between adult and older adult OT-QI performance and demographic and social characteristics (December 2025).

Variable	Category	Frail older adults *n*/*N* (%)	Univariate *P*-value	Frail older adults adjusted OR (95% CI)	Multivariable *P*-value	OT after stroke *n*/*N* (%)	Univariate *P*-value	OT after stroke adjusted OR (95% CI)	Multivariable *P*-value	OT after hip fracture *n*/*N* (%)	Univariate *P*-value	OT after hip fracture adjusted OR (95% CI)	Multivariable *P*-value
Gender	Male	2396/3101 (77.3)	.83	Reference	.790	1728/2966 (58.3)	.005	Reference	.070	1346/1884 (71.4)	.70	Reference	.530
	Female	1909/2463 (77.5)		1.02 (0.89–1.16)		1378/2218 (62.1)		1.12 (0.99–1.26)		2356/3275 (71.9)		0.96 (0.83–1.10)	
Age group (years)	65 to 74	1131/1484 (76.2)	.21	Reference	.800	919/1539 (59.7)	<.001	1.29 (1.09–1.52)	.003	808/1059 (76.3)	.001	1.04 (0.80–1.36)	.770
	75+	3174/4080 (77.8)		0.98 (0.85–1.14)		1530/2436 (62.8)		1.52 (1.30–1.78)	<.001	2366/3331 (71.0)		0.81 (0.64–1.03)	.090
SES	Low	520/724 (71.8)	<.001	Reference	.030	652/1050 (62.1)	.04	Reference	.060	674/932 (72.3)	.13	Reference	.003
	Low–medium	1262/1643 (76.8)		1.07 (0.86–1.34)	.56	911/1529 (59.6)		1.05 (0.86–1.26)	.65	1026/1436 (71.4)		1.09 (0.87–1.36)	.46
	Medium–high	1346/1742 (77.3)		0.97 (0.76–1.25)	.83	831/1445 (57.5)		1.01 (0.81–1.25)	.95	1093/1545 (70.7)		1.19 (0.93–1.52)	.17
	High	1067/1293 (82.5)		1.28 (0.98–1.68)	.07	590/943 (62.6)		1.27 (1.00–1.61)	.05	810/1083 (74.8)		1.53 (1.17–2.00)	.002
Ethnicity	Secular Jewish	3607/4601 (78.4)	<.001	Reference	.470	2275/3872 (58.8)	.001	Reference	<.001	2807/3944 (71.2)	.001	Reference	.020
	Arab	522/732 (71.3)		0.89 (0.71–1.11)	.290	725/1122 (64.6)		1.49 (1.23–1.81)	<.001	743/981 (75.7)		1.39 (1.10–1.75)	.006
	Orthodox Jewish	176/231 (76.2)		0.86 (0.62–1.20)	.380	106/190 (55.8)		0.96 (0.70–1.31)	.79	152/234 (65.0)		0.99 (0.72–1.36)	.960
Peripherality index	Peripheral	958/1474 (65.0)	<.001	Reference	<.001	990/1654 (59.9)	<.001	Reference	<.001	1088/1478 (73.6)	<.001	Reference	<.001
	Medium	1003/1212 (82.8)		2.70 (2.23–3.26)	<.001	711/1053 (67.5)		1.41 (1.19–1.67)	<.001	896/1142 (78.5)		1.35 (1.11–1.65)	.003
	Centre	2334/2867 (81.4)		2.32 (1.99–2.71)	<.001	1396/2459 (56.8)		0.92 (0.80–1.06)	.240	1705/2519 (67.7)		0.77 (0.65–0.91)	.002

Reference categories: male, age 45–64 years (stroke and hip fracture indicators) or 65–74 years (frail older adults indicator), low SES, secular Jewish, and peripheral regions. CI, confidence interval; OR, odds ratio; SES, socio-economic status.

#### Child development

For the *parental conversations* OT-QI, age group, SES, and peripherality index were significantly associated with indicator performance in the univariate analysis. In the multivariable model, children aged 6–9 years and those living in more peripheral areas had lower performance scores.

For the *treatment interruption* OT-QI, gender, SES, ethnicity, and peripherality index were significantly associated with interruption rates in the univariate analyses. In the multivariable model, significantly lower interruption rates were associated with higher SES groups and medium-peripherality areas.

#### Adults and older adults

For the *frail older adults* OT-QI, SES, ethnicity, and peripherality index were significantly associated with indicator performance in the univariate analyses. In the multivariable model, significantly higher performance scores were associated with medium and central areas compared with peripheral areas, while SES also remained significantly associated with performance.

For the *stroke* OT-QI, gender, age group, SES, ethnicity, and peripherality index were significantly associated with indicator performance in the univariate analyses. In the multivariable model, significantly higher performance scores were associated with older age groups, Arab participants compared with secular Jewish participants, and medium-peripherality areas compared with peripheral areas.

For the *hip fracture* OT-QI, age group, ethnicity, and peripherality index were significantly associated with indicator performance in the univariate analyses. In the multivariable model, significantly higher performance scores were associated with the highest SES group, Arab participants compared with secular Jewish participants, and medium-peripherality areas compared with peripheral areas.

## Discussion

### Statement of principle findings

This study describes the development, implementation, and 5-year monitoring of OT-QIs within CHS, Israel’s largest healthcare organization. The findings demonstrate the feasibility of embedding profession-specific QIs into routine organizational monitoring across diverse clinical populations and geographic regions. The OT-QIs were developed through a rigorous iterative process designed to support standardized quality monitoring in community-based OT services and may provide a practical model for other large healthcare organizations.

Over the monitoring period, most OT-QIs improved. In child development services, documented parental conversations increased, and treatment interruptions decreased. In geriatric services, OT provision for frail older adults increased substantially, while the stroke indicator showed a more modest increase. In contrast, the hip fracture indicator fluctuated and returned to approximately its initial level by December 2025, with no statistically significant overall change. Together, these findings illustrate the dynamic nature of OT-QI performance and the ongoing challenge of achieving consistent, equitable service delivery across populations and subgroups.

### Interpretation within the context of the wider literature

#### Child development services

The significant improvement in documented parental conversations (from 39.6% to 65.3%) aligns with the growing emphasis in paediatric rehabilitation on family-centred care rather than child-focused treatment [10–12]. Parental engagement enhances treatment outcomes, promotes skill generalization, and supports long-term developmental progress [26, 27]. The upward trend in our study also reflects organizational preference and efforts to strengthen family-centred practice.

Similarly, the significant parallel decline in treatment interruptions (from 8.1% to 3.7%) reflects improved continuity of care, which is essential for achieving functional goals [13, 14]. and may also improve service efficiency by reducing avoidable scheduling gaps. Although both child development QIs improved, subgroup patterns suggest that family engagement and treatment continuity remain shaped by age-related service structures, socio-economic context, and geographic accessibility.

#### Frail older adults OT-QI

The substantial increase in OT service delivery to frail older adults (from 51.8% to 77.4%) is clinically important given this population’s high risk of functional decline, falls, dependency, and institutionalization [22, 28]. This upward trend may reflect increased organizational awareness of OT’s role in geriatric care, improved identification and referral processes, expanded community-based capacity, and more consistent documentation of service delivery. However, subgroup analyses showed lower performance among those living in peripheral areas compared with those in medium and central areas, suggesting that geographic accessibility may remain a barrier despite overall improvement.

#### Stroke and hip fracture rehabilitation OT-QIs

Stroke performance showed a modest but statistically significant improvement (from 52.2% to 59.9%), suggesting improved yet still incomplete early OT service delivery following stroke. This early service delivery is an important factor in optimizing rehabilitation outcomes. Nevertheless, timely community-based OT after stroke remains an area requiring further service development and monitoring. The higher adjusted odds among older age groups may reflect differences in rehabilitation needs, referral patterns, clinical prioritization, or service-use behaviours.

The hip fracture indicator started with high performance (71.3%), declined during the monitoring period but returned to a similar level by December 2025 (71.76%), resulting in no statistically significant overall change. This pattern may reflect the 2024 expansion of the denominator definition and external service disruptions, although these explanations require cautious interpretation. Overall, these findings show that longitudinal QI performance is sensitive to operational definitions, documentation practices, and contextual disruptions.

In contrast, in child development services, the parental conversation QI increased, and treatment interruptions decreased, possibly reflecting greater flexibility in service delivery and strengthened continuity mechanisms. This result highlights that QI performance is sensitive to operational definitions, denominator changes, documentation practices, and external service disruptions. Such factors must be transparently documented when interpreting longitudinal QI trends. Nevertheless, implementation is not without challenges: Overemphasis on certain metrics and the added burden of documentation and administrative tasks can strain practitioners.

#### Demographic variables and quality indicators

Significant associations between OT-QI performance and socio-demographic/geographic variables suggest persistent inequities in service access and continuity. Lower performance in peripheral regions and lower SES groups is consistent with Israeli evidence on disparities in healthcare accessibility, particularly rehabilitation services [29]. The absence of gender differences may indicate relatively equitable provision of OT services across men and women within the healthcare system. Ethnic differences, mainly in stroke and hip fracture indicators, may reflect cultural, linguistic, or systemic barriers affecting rehabilitation use and follow-up care, consistent with previous reports of ethnic inequalities in healthcare utilization among older adults in Israel [30].

### Implications of OT-QIs for policy and service management

A central contribution of implementing OT-QIs was the creation of system-wide visibility for OT service delivery. Before 2021, OT performance monitoring at CHS was largely informal and decentralized. Standardized indicators enabled managers and clinicians to track trends, compare performance over time, and identify areas requiring intervention. Following implementation, CHS established national working groups for child development and adult OT services, where performance data are reviewed at clinic, district, and national levels and translated into targeted improvement strategies. Because OT-QIs are incorporated into CHS’s strategic quality framework, service managers are required to integrate them into annual work plans. In practice, these indicators support parent engagement, treatment continuity, timely OT provision for frail older adults, and coordination between hospital and community services after stroke or hip fracture. Together, these examples demonstrate how OT-QIs can generate actionable data for service management, resource allocation, and policy development.

### Limitations

Several limitations should be considered. First, the OT-QIs primarily capture process indicators related to service provision (e.g. whether services were delivered within a defined timeframe) rather than patient-level outcomes, such as functional improvement or daily participation, limiting conclusions regarding clinical effectiveness. Second, the absence of a comparison or control group precludes attributing observed performance changes solely to implementing OT-QIs, because concurrent organizational initiatives or system-level changes may have influenced the trends. Third, methodological modifications to the hip fracture indicator in 2024 (i.e. expanding the denominator definition) complicate longitudinal interpretation. Finally, indicator performance relies on EMR documentation; however, this study did not distinguish improved documentation from actual changes in service provision.

### Future research directions

Future research should link process-based OT-QIs to patient-level functional, participation, and quality-of-life outcomes; examine whether OT-QIs can detect and reduce inequities in service access and continuity; and explore implementation mechanisms, barriers, and facilitators from the perspectives of patients, families, clinicians, and managers.

## Conclusions

This study demonstrates the feasibility and value of developing and implementing OT-specific QIs within a large, publicly funded healthcare organization. The findings show that profession-specific QIs can be embedded in routine EMR-based monitoring systems to track service delivery across paediatric, geriatric, orthopaedic, and neurological rehabilitation services. Most indicators improved over time, suggesting progress in family engagement, treatment continuity, and access to OT services for key populations; the stable hip fracture indicator underscores the need for ongoing refinement of operational definitions and monitoring procedures.

Beyond monitoring overall performance, the OT-QIs reveal meaningful socio-demographic and geographic variations, indicating their potential role in identifying service delivery inequities. These findings support the use of OT-QIs as practical tools for service management, accountability, and quality improvement in rehabilitation.

## Supplementary Material

mzag081_Supplementary_Data

## Data Availability

Data underlying this study are not publicly available due to institutional restrictions but may be available upon reasonable request to the corresponding author and subject to Clalit Health Services approval.
