# Supplementary material for: Implementing occupational therapy quality indicators in a large healthcare system: lessons from a 5-year performance monitoring study
Source: Int J Qual Health Care. 2026 Jun 5;38(2):mzag081. doi: 10.1093/intqhc/mzag081 (PMC13285999; doi:10.1093/intqhc/mzag081)
Supplement: mzag081_Supplementary_Data [file mzag081_supplementary_data.docx]

**Table S1.** Longitudinal Occupational Therapy (OT) Quality Indicator (OT-QI) Performance Data Across All Measurement Periods (January 2021 to December 2025)

| **OT-QI** | **1/2021** | **2/2021** | **3/2021** | **4/2021** | **5/2021** | **6/2021** | **7/2021** | **8/2021** | **9/2021** | **10/2021** | **11/2021** | **12/2021** |
| --- | --- | --- | --- | --- | --- | --- | --- | --- | --- | --- | --- | --- |
| Parental conversations | 39.6% | 40.1% | 39.9% | 40.9% | 41.9% | 43.9% | 45.1% | 45.4% | 46.9% | 48.6% | 50.4% | 51.4% |
| Treatment interruption | 8.1% | 9.2% | 9.9% | 10.6% | 11.1% | 10.6% | 10.4% | 10.2% | 9.7% | 9.2% | 7.6% | 7.1% |
| Frail older adults OT | 51.8% | 52.4% | 54.4% | 55.1% | 55.5% | 55.2% | 55.3% | 56.1% | 56.1% | 56.2% | 56.0% | 56.6% |
| Stroke | 52.2% | 52.7% | 53.4% | 54.0% | 55.1% | 55.7% | 56.1% | 56.5% | 56.7% | 56.8% | 57.2% | 57.7% |
| Hip fracture | 71.3% | 71.2% | 71.2% | 71.7% | 73.0% | 73.7% | 74.4% | 61.3% | 61.9% | 62.1% | 62.3% | 62.5% |
|  | **1/2022** | **2/2022** | **3/2022** | **4/2022** | **5/2022** | **6/2022** | **7/2022** | **8/2022** | **9/2022** | **10/2022** | **11/2022** | **12/2022** |
| Parental conversations | 51.7% | 52.3% | 52.6% | 52.4% | 52.8% | 53.6% | 53.8% | 53.6% | 53.6% | 53.0% | 53.3% | 52.8% |
| Treatment interruption | 7.2% | 7.8% | 7.6% | 7.8% | 8.4% | 8.6% | 8.8% | 8.8% | 8.8% | 7.7% | 6.7% | 6.1% |
| Frail older adults OT | 56.1% | 56.2% | 55.6% | 56.1% | 57.0% | 57.2% | 57.5% | 58.4% | 58.9% | 58.1% | 58.1% | 58.5% |
| Stroke | 57.1% | 57.6% | 57.5% | 57.7% | 57.9% | 58.1% | 58.0% | 57.5% | 57.4% | 57.2% | 57.2% | 57.1% |
| Hip fracture | 63.1% | 62.9% | 63.1% | 63.1% | 63.0% | 63.1% | 63.1% | 63.0% | 62.7% | 62.9% | 63.2% | 62.9% |
|  | **1/2023** | **2/2023** | **3/2023** | **4/2023** | **5/2023** | **6/2023** | **7/2023** | **8/2023** | **9/2023** | **10/2023** | **11/2023** | **12/2023** |
| Parental conversations | 54.0% | 54.5% | 55.0% | 55.8% | 55.7% | 56.5% | 56.7% | 57.1% | 58.6% | 60.0% | 60.1% | 60.5% |
| Treatment interruption | 6.2% | 6.5% | 6.6% | 6.7% | 6.9% | 6.7% | 6.5% | 6.3% | 6.1% | 5.9% | 5.2% | 5.0% |
| Frail older adults OT | 58.5% | 59.1% | 59.6% | 61.0% | 60.9% | 60.6% | 60.7% | 61.2% | 61.1% | 61.3% | 61.7% | 60.8% |
| Stroke | 57.0% | 57.1% | 57.6% | 57.4% | 57.1% | 56.8% | 57.0% | 57.0% | 56.6% | 56.7% | 56.0% | 55.6% |
| Hip fracture | 63.0% | 62.7% | 62.3% | 61.7% | 60.9% | 60.6% | 60.6% | 60.3% | 59.4% | 58.8% | 58.4% | 56.8% |
|  | **1/2024** | **2/2024** | **3/2024** | **4/2024** | **5/2024** | **6/2024** | **7/2024** | **8/2024** | **9/2024** | **10/2024** | **11/2024** | **12/2024** |
| Parental conversations | 60.2% | 60.2% | 59.9% | 60.1% | 60.2% | 59.9% | 60.1% | 60.2% | 60.6% | 60.6% | 61.8% | 62.5% |
| Treatment interruption | 4.4% | 3.9% | 4.1% | 4.4% | 4.6% | 4.5% | 4.3% | 4.4% | 4.2% | 4.3% | 3.8% | 3.8% |
| Frail older adults OT | 66.2% | 66.1% | 65.3% | 65.6% | 66.8% | 67.4% | 68.6% | 69.2% | 70.7% | 71.0% | 73.6% | 74.6% |
| Stroke | 50.5% | 50.4% | 50.1% | 50.2% | 50.5% | 52.4% | 53.2% | 53.1% | 53.2% | 53.8% | 54.2% | 54.7% |
| Hip fracture | 51.6% | 51.6% | 51.6% | 50.7% | 50.6% | 66.4% | 66.6% | 66.8% | 66.7% | 66.9% | 67.1% | 67.3% |
|  | **1/2025** | **2/2025** | **3/2025** | **4/2025** | **5/2025** | **6/2025** | **7/2025** | **8/2025** | **9/2025** | **10/2025** | **11/2025** | **12/2025** |
| Parental conversations | 63.3% | 63.7% | 63.9% | 64.3% | 64.9% | 65.0% | 64.5% | 64.1% | 63.9% | 63.8% | 64.6% | 65.3% |
| Treatment interruption | 3.6% | 3.7% | 3.8% | 3.8% | 4.1% | 4.1% | 4.1% | 4.2% | 3.7% | 3.8% | 3.8% | 3.7% |
| Frail older adults OT | 76.1% | 77.4% | 78.1% | 78.7% | 78.3% | 77.6% | 77.6% | 77.1% | 77.0% | 76.8% | 77.3% | 77.4% |
| Stroke | 59.4% | 59.2% | 59.3% | 59.4% | 59.6% | 59.8% | 59.5% | 59.7% | 59.9% | 59.9% | 60.1% | 59.9% |
| Hip fracture | 70.9% | 71.0% | 70.8% | 70.9% | 71.1% | 71.3% | 71.5% | 71.9% | 72.1% | 72.0% | 72.0% | 71.8% |

**Table S2**. Occupational Therapy Quality Indicator Performance by Age Group Across Measurement Periods, January 2021 to December 2025

| **OT-QI** | **Age group (years)** | **Target Population (12/2025)** | **Jan-2021** | **June-2021** | **Dec-2021** | **June-2022** | **Dec-2022** | **June-2023** | **Dec-2023** | **June-2024** | **Dec-2024** | **Jun-2025** | **Dec-2025** | **Relative change (12/2025 to 01/2021)** |
| --- | --- | --- | --- | --- | --- | --- | --- | --- | --- | --- | --- | --- | --- | --- |
|  |  |  | **Percentage** | | | | | | | | | | | |
| Parental conversations | All | 3991 | 39.6 | 43.9 | 51.4 | 53.6 | 52.8 | 56.5 | 60.5 | 59.9 | 62.5 | 65.0 | 65.3 | 64.7 |
|  | 0–2 | 608 | 44.6 | 41.1 | 49.1 | 50.0 | 56.3 | 59.1 | 63.0 | 61.4 | 65.1 | 64.8 | 66.9 | 50.1 |
|  | 3–5 | 2974 | 40.3 | 45.2 | 53.3 | 55.8 | 53.5 | 57.8 | 61.3 | 60.5 | 63.0 | 65.5 | 66.2 | 64.3 |
|  | 6–9 | 409 | 31.1 | 39.4 | 41.8 | 49.1 | 44.3 | 46.6 | 52.0 | 54.3 | 55.9 | 62.3 | 56.2 | 80.6 |
| Treatment interruption | All | 3991 | 8.1 | 10.6 | 7.1 | 8.6 | 6.1 | 6.7 | 5.0 | 4.5 | 3.8 | 4.1 | 3.7 | -54.7 |
|  | 0–2 | 608 | 9.5 | 11.8 | 10.2 | 9.8 | 6.7 | 6.7 | 4.3 | 4.6 | 5.3 | 4.6 | 2.5 | -73.9 |
|  | 3–5 | 2,974 | 8.1 | 10.6 | 6.7 | 8.7 | 6.4 | 7.0 | 5.2 | 4.7 | 3.8 | 4.3 | 4.1 | -49.5 |
|  | 6–9 | 409 | 6.9 | 9.0 | 5.3 | 7.7 | 3.9 | 4.9 | 4.4 | 3.4 | 2.0 | 2.8 | 2.4 | -64.4 |
| Frail older adults | All | 5564 | 51.8 | 55.2 | 56.6 | 57.2 | 58.5 | 60.6 | 60.8 | 67.4 | 74.6 | 77.6 | 77.4 | 49.4 |
|  | 65–74 | 1584 | 54.8 | 57.6 | 58.7 | 60.1 | 59.2 | 61.3 | 60.9 | 68.6 | 75.2 | 75.3 | 75.9 | 38.6 |
|  | 75+ | 3980 | 50.0 | 53.8 | 55.4 | 55.9 | 58.2 | 60.2 | 60.7 | 66.8 | 74.4 | 78.5 | 77.9 | 55.9 |
| Stroke | All | 5184 | 52.2 | 55.7 | 57.7 | 58.1 | 57.1 | 56.8 | 55.6 | 52.4 | 54.7 | 59.8 | 59.9 | 14.8 |
|  | 00–44 | 146 | 32.4 | 45.8 | 52.9 | 62.4 | 60.9 | 56.4 | 50.0 | 47.3 | 46.2 | 51.0 | 54.8 | 69.1 |
|  | 45–64 | 1050 | 54.5 | 56.3 | 53.7 | 52.7 | 51.5 | 50.8 | 52.6 | 48.8 | 50.8 | 56.0 | 54.2 | -0.6 |
|  | 65+ | 3988 | 52.6 | 56.1 | 59.2 | 59.5 | 58.6 | 58.5 | 56.6 | 53.6 | 56.1 | 61.1 | 61.6 | 17.1 |
|  | 65–74 | 1523 | 52.6 | 56.1 | 57.8 | 57.0 | 55.5 | 55.1 | 53.5 | 52.1 | 53.6 | 60.0 | 59.8 | 13.6 |
|  | 75+ | 2465 | 52.5 | 56.2 | 60.4 | 61.0 | 60.6 | 60.5 | 58.5 | 54.5 | 57.6 | 61.8 | 62.8 | 19.5 |
| Hip fracture | All | 5159 | 71.3 | 73.7 | 62.5 | 63.1 | 62.9 | 60.6 | 56.8 | 66.4 | 67.3 | 71.3 | 71.8 | 0.6 |
|  | 00–44 | 294 | 63.7 | 61.2 | 63.0 | 58.4 | 61.3 | 59.1 | 52.5 | 52.0 | 49.8 | 54.5 | 57.1 | -10.3 |
|  | 45–64 | 466 | 79.9 | 78.5 | 73.0 | 73.5 | 73.2 | 70.7 | 68.8 | 72.7 | 74.1 | 75.1 | 75.3 | -5.7 |
|  | 65+ | 4399 | 70.5 | 73.9 | 61.0 | 62.2 | 61.7 | 59.6 | 55.7 | 66.7 | 67.8 | 72.1 | 72.4 | 2.6 |
|  | 65–74 | 1045 | 73.7 | 78.0 | 66.1 | 68.8 | 68.7 | 68.2 | 64.1 | 70.3 | 72.3 | 76.0 | 76.7 | 4.1 |
|  | 75+ | 3354 | 69.1 | 72.4 | 59.0 | 60.4 | 59.6 | 57.0 | 53.2 | 65.5 | 66.4 | 70.8 | 71.0 | 2.7 |
